# Tissue-Resident Macrophages in Fungal Infections

**DOI:** 10.3389/fimmu.2017.01798

**Published:** 2017-12-12

**Authors:** Shengjie Xu, Mari L. Shinohara

**Affiliations:** ^1^Department of Immunology, Duke University School of Medicine, Durham, NC, United States; ^2^Department of Molecular Genetics and Microbiology, Duke University School of Medicine, Durham, NC, United States

**Keywords:** tissue-resident macrophages, fungal infections, microglia, alveolar macrophages, *Candida*, *Cryptococcus*, *Aspergillus*

## Abstract

Invasive fungal infections result in high morbidity and mortality. Host organs targeted by fungal pathogens vary depending on the route of infection and fungal species encountered. *Cryptococcus neoformans* infects the respiratory tract and disseminates throughout the central nervous system. *Candida albicans* infects mucosal tissues and the skin, and systemic *Candida* infection in rodents has a tropism to the kidney. *Aspergillus fumigatus* reaches distal areas of the lung once inhaled by the host. Across different tissues in naïve hosts, tissue-resident macrophages (TRMs) are one of the most populous cells of the innate immune system. Although they function to maintain homeostasis in a tissue-specific manner during steady state, TRMs may function as the first line of defense against invading pathogens and may regulate host immune responses. Thus, in any organs, TRMs are uniquely positioned and specifically programmed to function. This article reviews the current understanding of the roles of TRMs during major fungal infections.

## Introduction

Macrophages were initially discovered in the late nineteenth century by Metchnikoff and named for its phagocytic activity as “devouring cells” in Greek (1, 2). They are capable of engulfing and digesting cellular debris, foreign substances, and microorganisms, which are critical for tissue remodeling and immune defense against pathogens. Based on the morphology, function, origin, and kinetics of these phagocytes, macrophages were categorized into the “mononuclear phagocytes system (MPS)” (3). Even after a century since the discovery of macrophages, research efforts have continuously focused on the origins and functions of macrophages for their significant impact on tissue homeostasis and disease pathogenesis.

Tissue-resident macrophages (TRMs) consist of heterogeneous subsets of macrophages distributed in tissues across the body and contribute to tissue homeostasis and immunosurveillance (4, 5). Depending on which organs they reside, some TRMs have specific names, such as alveolar macrophages (AMs) (lung), microglia (brain), Kupffer cells (liver), renal macrophages (kidney), and osteoclasts (skeletal system). As such specific names indicate, TRMs are considered to have specific functions due to various tissue microenvironments (6, 7). This mini-review provides an outline of several major TRMs in fungal infections, mainly focusing on murine studies, by which a majority of mechanistic insights about TRMs have been obtained.

## Origins of TRMs

### Developmental Origins of TRMs

Tissue-resident macrophages used to be considered as cells derived from circulating monocytes during the early establishment of the MPS (3). However, a series of recent studies drastically changed this notion, particularly through the technical advancement of *in vivo* cellular lineage-tracing by employing the “fate-mapping” technique using the mouse Cre-lox genetic system. Such *in vivo* lineage-tracing approaches have shown, for example, that microglia arise early in mouse development and are derived from primitive macrophages in the yolk sac (YS) (8). These studies suggested that microglia are ontogenically distinct from monocyte-derived macrophages (MDMs), which are of the hematopoietic origin. In addition to microglia, F4/80^hi^ Kupffer cells and epidermal Langerhans cells were demonstrated to be YS-derived and do not require Myb, a transcription factor required for the development of hematopoietic stem cells (HSCs) (9). By employing the conditional CX_3_CR1 fate-mapping system, another study showed that origins of Kupffer cells, AMs, splenic, and peritoneal macrophages, are also embryonic, at least in part (10). Introduction of fate-mapping markers other than CX_3_CR1 further clarified that TRMs in many tissues consist of mixed populations of the embryonic (YS and/or fetal liver) and the BM hematopoietic origins, except for microglia that are exclusively of the YS-origin (11–13).

A majority of TRMs are self-maintained throughout adult life with minimal contribution from circulating monocytes (14). However, populations of TRMs can also be replaced. For example, intestinal macrophages in mouse neonates are derived from YS and fetal liver, but do not persist into adulthood and are replaced by MDMs around the time of weaning (15). Cardiac macrophages are established from YS and fetal monocyte progenitors, but disruption of homeostasis replaces the population with MDMs (11). These murine studies strongly suggested that TRMs, in general, are derived from diverse precursors including YS macrophages, fetal liver monocytes, and even circulating HSC-derived monocytes; and ontogenic origins of TRMs greatly vary depending on tissues.

### TRMs Reflecting Organ-Specific Microenvironments

Tissue-resident macrophages develop locally and adapt to tissue microenvironments during embryogenesis and beyond. Distinct gene expression patterns were identified among local TRMs from various tissues (6, 7, 16, 17), and are often reflected at the epigenetic level, particularly indicated by differential histone marks on the enhancer landscape (6, 7). Multiple pieces of evidence have suggested that such tissue-specific patterns of gene expression in TRMs are influenced by tissue-specific environmental factors, including heme (18), retinoic acid (6, 17), and TGF-β (6, 19). Interestingly, macrophage “precursors” derived from YS, fetal liver, and adult monocytes appear to have the plasticity to become certain TRMs, based on tissue-specific gene expression profiles. For example, macrophages precursors from various origins develop into functional and self-maintaining AMs, when transplanted to an empty alveolar niche (20). However, once differentiated into organ-specific macrophages, TRMs, except for Kupffer cells, cannot efficiently colonize the empty AM niche (20), suggesting that the plasticity would be lost after the precursor stage. Thus, functions of TRMs are actively shaped by their local tissue microenvironment.

## TRMs in Antifungal Responses

Critical steps to protect hosts from infections include; early recognition of the fungi, activation of host immunity, and killing of the spores and vegetative fungal cells to contain fungal dissemination (21–24). During early stages of fungal infections, infected hosts rely on tissue-resident “cells,” not necessarily TRMs alone, to function as the first line of defense. Here, despite the tissue-specific functions of TRMs from various organs, a general expectation for TRMs is to function as immune sentinels to detect infections at the front line. In fact, TRMs express a wide array of cell surface receptors that sense intruding microbes and produce chemokines and cytokines to recruit and activate other cell subsets for further help (25, 26). However, do TRMs always work to protect hosts? We will visit this topic in the following subsections. As some backgrounds for this section, we would like to mention that TRMs are not considered to play a role in T cell priming with microbe-derived antigens in draining lymph nodes because they are not migratory cells (27). It is also of note that CCR2^+^ inflammatory MDMs play critical role in fungal clearance (28–32). Here, CCR2^+^ MDMs are recruited from circulation by chemoattractants secreted by sentinel cells. In the following subsections and Table 1, we focus on the early interaction of TRMs with fungi.

**Table 1:** Tissue-resident macrophage antifungal response.

Cell type	Fungal pathogen	Macrophage responses	Experimental approaches
Alveolar macrophages	*C. neoformans*	Take up spores which are opsonized by complement and antibodies through CR3 and Fcγ receptors (33, 34) mediated by S1PR2 (35)	Stimulating adherent murine bronchoalveolar lavage (BAL) cells in tissue culture (35)
		Produce TNFα, CXCL1, CXCL2, CCL2, and IL-1β (36)	Stimulating adherent murine BAL cells in tissue culture (36)
	*A. fumigatus*	Trap *A. fumigatus* conidia by pseudopods and take up conidia in actin-dependent manner (37)	Stimulating MH-S murine AM cell line in tissue culture (38)
		Kill *A. fumigatus* conidia through phagolysosome acidification (37) and production of reactive oxidant species (39)	Stimulating adherent murine BAL cells in tissue culture (39)
		Bind and uptake *A. fumigatus* conidia through SP-A and SP-D (40)	Stimulating BAL cells from aspergillosis patients in tissue culture (40)
		Detect cell wall β-1,3-glucans through Dectin-1 (41) and NOD2 receptors (42)	Murine *A. fumigatus* pulmonary infection model: AMs identified as CD45^+^Ly6G^−^ CD11c^+^ cells (41)
		Induce proinflammatory cytokines and chemokines (38)	Stimulating MH-S cell line and adherent murine BAL cells in tissue culture (38)
Microglia	*C. albicans*	Detect cell wall β-glucan through Dectin-1 and activate Syk, Vav1, and PI3K (43) required for phagocytosis (44), and superoxide production (45)	Stimulating murine microglia primary cell culture and BV-2 microglial cell line (43)
		Increase CD11b expression and change morphology (46)	Invasive murine candidiasis model: evaluating MHC-II^+^Iba1^+^ microglia *in situ* or CD45^+^CD11b^+^MHC-II^+^ microglia by flow cytometry (46)
	*C. neoformans*	Produce chemokines, MCP-1, MIP-1α, and MIP-1β through FcR-mediated activation by antibody-bound *C. neoformans* (47)	Stimulating human fetal microglia cells with *C. neoformans* and antifungal antibodies in tissue culture (47)
		Require IFNγ or LPS priming to eliminate *C. neoformans* through nitric oxide production (48)	Stimulating BV-2 microglial cell line (48)
	*A. fumigatus*	Require complement system for phagocytosis of *A. fumigatus* conidia (49, 50)	Stimulating human primary microglia in tissue culture (50)
Kidney macrophages	*C. albicans*	Internalize conidia and pseudohyphae (29)	Invasive murine candidiasis model: evaluating CX3CR1^+^ renal macrophages *in situ* (29)
		Produce chemokines, CXCL1, and CXCL2 (25)	Invasive murine candidiasis model: evaluating SSC^lo^CD45^+^Ly6G^−^CD11b^+^F4/80^hi^ renal macrophages by flow cytometry (25)

### Lung—AMs

Because lungs are exposed to the outer environment, they constantly inhale microbes, which enter the distal airway to bronchioles and alveoli. AMs are lung-resident macrophages considered to be largely derived from fetal liver monocytes (10, 13, 51) and represent more than 90% of leukocytes in a bronchoalveolar lavage in healthy animals (52). Since fungal infections through the pulmonary route have been intensively studied, AMs may be the best-documented TRMs in fungal infections. Here, we discuss AMs and two major pulmonary fungal pathogens, *Cryptococcus neoformans* and *Aspergillus fumigatus*, which can cause serious invasive cryptococcosis and aspergillosis, respectively (21, 53–55).

*C. neoformans* spores and *A. fumigatus* conidia enter into the lungs by inhalation and encounter lung-resident cells first, including AMs. Although AMs are not effective in antigen presentation to T cells due to their low level of costimulatory molecules (56), AMs are considered to be at the first line of immune defense against pulmonary pathogens (57). AMs express complement receptor 3 (CR3) and Fcγ receptors (FcγR) to opsonize and phagocytose *C. neoformans* spores (33, 34, 58, 59). Phagocytosis of *C. neoformans* spores is enhanced by extracellular sphingosine-1-phosphate, which upregulates FcγR expression on AMs (35). In *A. fumigatus* infection, AMs can trap dormant *A. fumigatus* conidia with pseudopods and endocytose conidia in an actin-dependent manner (37, 60). Although neutrophils are the main population involved in complement-dependent opsonization, phagocytosis, and killing of the fungi (61, 62), AMs can also kill internalized *A. fumigatus* conidia by detection of conidia swelling and the endosome–phagosome fusion, resulting in acidification of the organelles (37). Activation of NADPH oxidase in AMs was also reported (39), suggesting AMs to gain an “M1” phenotype. Alternatively, another study showed that *A. fumigatus* infection promotes AMs to gain an alternative activated macrophage phenotype, or also known as the M2 phenotype, based on upregulation of M2 macrophage markers, such as gene transcripts encoding arginase-1 (Arg1), Ym1, and CD206 (63). Interestingly, the study did not observe the induction of *Nos2*, a major M1 macrophage marker (63). It was suggested that Arg1-expressing AMs potentially deprive L-arginine, a substrate of arginase. Since L-arginine is an essential nutrient source of fungi, the expression of Arg1 may result in inhibiting fungal growth through arginine deprivation (63). These studies suggested the presence of multiple mechanisms by which AMs protects hosts from fungal infections.

Failure in the initial clearance of invaded fungi allows them to take advantage of the humid and nutrient-rich milieu in the lung to disseminate. As the next layer to contain fungal dissemination, inflammatory neutrophils and monocytes need to be recruited in the lung. Here, it is possible that AMs play a sentinel role to recruit such inflammatory cells by secreting cytokines and chemokines to fight against fungi. For example, dectin-1 on AMs detects β-glucans on the fungal cell surface (41, 64) and stimulates the production of proinflammatory cytokines TNFα, IL-6, and IL-18 (65). Intracellular receptor NOD2 in AMs can also induce the synthesis of cytokines, such as IL-12, IFN-γ, GM-CSF, CCL2/MCP-1, CXCL2/MIP-2, and CXCL1/KC (38, 42). It is of note that the majority of these studies on cytokine and chemokine expression were performed with isolated AMs or cell lines in tissue culture. Thus, *in vivo* protein expression patterns of AMs to *A. fumigatus* and *C. neoformans* infections need to be studied.

Neutrophil chemoattractants, such as CXCL1 and CXCL2, have a great impact on the host protection from *A. fumigatus* infection (66, 67), and the main source of the chemoattractants in *A. fumigatus* infection was reported to be epithelial cells, rather than AMs (68). Indeed, AM depletion by clodronate does not alter neutrophil recruitment and host mortality in pulmonary *A. fumigatus* infection (69). Thus, a role of AMs in *A. fumigatus* may be minor. In contrast in *C. neoformans* infection, AMs highly express CXCL1 and CXCL2, as well as TNFα (36), but *C. neoformans* can survive in AMs and contribute to latent infection (70). However, it is puzzling that depletion of AMs and DCs “together,” by using CD11c-DTR mice (AMs and DCs are CD11c positive), resulted in more neutrophil infiltration in the lung 4 days after *C. neoformans* infection and enhanced mortality with severe lung inflammation (71). Although it is not clear which cell type, DCs, or AMs, is dominant in inhibiting neutrophil recruitment in the lung, questions that can be brought up are how DCs and/or AMs inhibit neutrophil recruitment and whether the inhibition occurs only under some conditions. Since it is technically difficult to deplete AMs alone, we still need to wait to understand if and how AMs are detrimental or protective in fungal infections.

### Central Nervous System (CNS)—Microglia

Fungal infections in the CNS are usually secondary to infections in peripheral tissues. Yet, once fungal pathogens reach to the CNS, it can be fatal to hosts. Some species of *Candida, Cryptococcus*, and *Aspergillus* can cause life-threatening CNS infections in immunocompromised patients (72–74). Microglia reside in the CNS parenchyma and are poised to provide the first line of defense against invading pathogens. Through the expression of various pattern-recognition receptors, microglia can recognize a wide range of pathogens that colonize the CNS (75, 76). In this section, we discuss responses of microglia during CNS infection by these fungi.

*Candida albicans* commonly colonizes the mucocutaneous locations in the host, and can also invade the bloodstream to cause systemic candidiasis. Innate immunity is the dominant protective mechanism against disseminated candidiasis. Microglia detect β-glucans through dectin-1, resulting in phosphorylation of Syk (43), and activation of Vav1 and PI3K, which are required for phagocytosis and superoxide production (45). However, dectin-1 stimulation alone is not sufficient for microglia to induce cytokines or chemokine production (43). This suggests a unique mechanism of dectin-1 signaling in microglia distinct from other types of TRMs and MDMs, in which dectin-1 signaling is sufficient for production of cytokines and chemokines. Microglia are also found in the retina and activated by invasive candidiasis, resulting in enhanced expression of cell surface CD11b, and morphological change (46), as well as phagocytosis of *C. albicans* conidia through dectin-1 activation (44).

In contrast to *Candida, C. neoformans* spores are not effectively cleared by microglia. Thus, microglia require other immune cells and mechanisms to effectively combat *C. neoformans* infection in the CNS (77, 78). Opsonization of *C. neoformans* spores by antibodies plays a critical role in the induction of cytokine and chemokine expression in microglia (48). For example, opsonizing antibodies induce microglial expression of chemokines, such as CCL2/MCP-1, CCL3/MIP-1α, and CCL4/MIP-1β, but the response is also known to be inhibited by cryptococcal capsular polysaccharides (47). In addition to antibodies, LPS and IFNγ promote the killing of opsonized and unopsonized *C. neoformans* by augmenting nitric oxide production without inducing phagocytosis in a microglial cell line (48). Another study showed that IFNγ is required for enhanced anticryptococcal responses when microglia are activated by intracranial injection of IL-2 and a CD40 agonistic antibody (79). Taken together, IFNγ appears to be critical for microglia to respond to *C. neoformans*.

*Aspergillus fumigatus* also causes meningitis, but little is known about responses of microglia to *A. fumigatus*. One study showed that CR3 expression of microglia is reduced by an *A. fumigatus-*derived protease, resulting in a significant decrease in phagocytosis by primary human microglia (49). The high frequency of host mortality by cerebral aspergillosis suggests that antifungal responses of microglia are not efficient, although it might be possible that IFNγ also enhances the response against *Aspergillus* by microglia.

Taken together, these studies suggest that microglia are not efficient in fungal clearance. Although it is not clear why microglia are not effective cells among the MPS, the specific microenvironment of the CNS, which is known as an immune-privileged site, may be involved in shaping the character of microglia. The CNS is isolated from other peripheral organs because it is separated from blood circulation by the blood–brain barrier. The physical separation of the CNS from the immune system in the rest of organs, at least in part, may contribute to the specific development and functions of microglia, distinct from the rest of TRMs.

### Kidney—Renal Macrophages

In healthy kidneys, immune cells are rarely found except for resident DCs and macrophages (80). Renal macrophages are found in the tubulointerstitium (81), a compartment of the kidney bounded by the vasculature and nephrons, and comprising about 80% of kidney volume (80). Renal macrophages in adult mice are largely derived from fetal liver monocytes (11, 82) and have been extensively studied due to their involvement in immune homeostasis (83–85) and host defense against infections (29, 86).

The kidney is a main target organ in murine systemic candidiasis (87, 88), but not necessarily a primary target in human systemic candidiasis (89). Nevertheless, host resistance heavily depends on the immune system in the kidney. For example, renal macrophages, as well as possibly splenic and liver macrophage, are considered to be protective in host defense against *Candida* (29, 87, 90). CX_3_CR1-deficient mice are susceptible to *Candida* infection, possibly due to reduced numbers of kidney-resident and -infiltrated macrophages (91). As early as 2 h after *Candida* infection, renal macrophages elicit their protective responses by internalizing conidia and encasing pseudohyphal elements (91). In addition to their phagocytic ability, renal macrophages isolated from naïve mice are shown to kill *Candida* conidia in tissue culture (91). Besides their endogenous fungal-killing ability, kidney F4/80^hi^ macrophages also recruit neutrophils by secreting high levels of chemokine CXCL2 in the first 24 h of systemic *Candida* infection in an autophagy-dependent manner (25), indeed playing a role as immune sentinels. In summary, kidney macrophages are important players in fungal clearance in murine candidiasis model.

## Closing Remarks

Our knowledge on TRMs identities and functions has been greatly expanded in the last decade. Depending on the physical locations and fungal pathogens, TRMs respond in different ways. Tissue-specific factors may also have impacts on the antifungal outcome of TRMs. However, there are still many unanswered questions and technical hurdles to further advance the field. Here, we close our discussion with six questions.

(A) *Do the functions of TRMs from various organs share something in common?* Because TRMs are shaped by tissue-specific environments to acquire unique intracellular gene expression profile and assisted by tissue factors to enhance their antifungal response, previous studies have focused on the dissimilarity among TRMs from various organs. Yet, all TRMs are expected to play a similar role in maintaining immune surveillance and behaving as sentinels when infections occur. Thus, despite their organ-specific environments, TRMs could potentially share some functions, particularly as sentinels during infections. (B) *To which extent can result from tissue culture experiments be applied to TRMs’ functions in vivo?* Majority of functional studies on TRMs have been performed in tissue culture or even with cell lines. It is not clear if *ex vivo* behaviors of TRMs reflect those *in vivo*. (C) *Do human TRMs behave similarly to murine TRMs?* Due to the technical limits to isolate TRMs from humans, a majority of TRM studies have been carried out by using animals. Therefore, it is again not clear if and to what extent TRMs from human and murine share similar responses. (D) *Are TRMs involved in allowing fungi to switch from commensal/non-pathogenic to pathogenic?* TRMs’ involvement in the switching might be possible because of the localization of TRMs in tissues where commensal fungi are homed. (E) *Are TRMs heterogeneous if they are within a single organ?* For example, the presence of microglia subsets has been identified (92, 93). It is intriguing to explore possible cellular subsets within TRMs in a single tissue and their possibly distinct functions. To answer the question, new technologies, such as single-cell sequencing or CyTOF would be very powerful tools to answer the question. (F) *How can we “specifically” deplete a certain TRM population?* One of the most significant technical challenges in studying TRMs may be depleting a certain population of TRMs. Clodronate-liposome is used to deplete TRMs, but it is not specific depletion. There are genetically modified mice and antagonists of certain receptors used to particularly deplete microglia. However, what is the best method to deplete AMs or Kupffer cells, for example? These are at least several questions and challenges to overcome to better understand TRMs in fungal infections and even other pathogenic conditions.

## Author Contributions

All authors listed have made a substantial, direct, and intellectual contribution to the work and approved it for publication.

## Conflict of Interest Statement

The authors declare that the research was conducted in the absence of any commercial or financial relationships that could be construed as a potential conflict of interest.
